# The Role of Mst1 in Lymphocyte Homeostasis and Function

**DOI:** 10.3389/fimmu.2018.00149

**Published:** 2018-02-05

**Authors:** Jiali Cheng, Yukai Jing, Danqing Kang, Lu Yang, Jingwen Li, Ze Yu, Zican Peng, Xingbo Li, Yin Wei, Quan Gong, Richard J. Miron, Yufeng Zhang, Chaohong Liu

**Affiliations:** ^1^Department of Microbiology, School of Basic Medicine, Tongji Medical College, Huazhong University of Science and Technology, Wuhan, China; ^2^Wuhan Children’s Hospital, Tongji Medical College, Huazhong University of Science and Technology, Wuhan, China; ^3^Department of Immunology, School of Medicine, Yangtze University, Jingzhou, China; ^4^Clinical Molecular Immunology Center, School of Medicine, Yangtze University, Jingzhou, China; ^5^State Key Laboratory Breeding Base of Basic Science of Stomatology (Hubei-MOST) and Key Laboratory of Oral Biomedicine Ministry of Education, School and Hospital of Stomatology, Wuhan University, Wuhan, China

**Keywords:** hippo, lymphocyte, migration, proliferation, apoptosis

## Abstract

The Hippo pathway is an evolutionarily conserved pathway crucial for regulating tissue size and for limiting cancer development. However, recent work has also uncovered key roles for the mammalian Hippo kinases, Mst1/2, in driving appropriate immune responses by directing T cell migration, morphology, survival, differentiation, and activation. In this review, we discuss the classical signaling pathways orchestrated by the Hippo signaling pathway, and describe how Mst1/2 direct T cell function by mechanisms not seeming to involve the classical Hippo pathway. We also discuss why Mst1/2 might have different functions within organ systems and the immune system. Overall, understanding how Mst1/2 transmit signals to discrete biological processes in different cell types might allow for the development of better drug therapies for the treatments of cancers and immune-related diseases.

## Introduction

**Figure 1:**
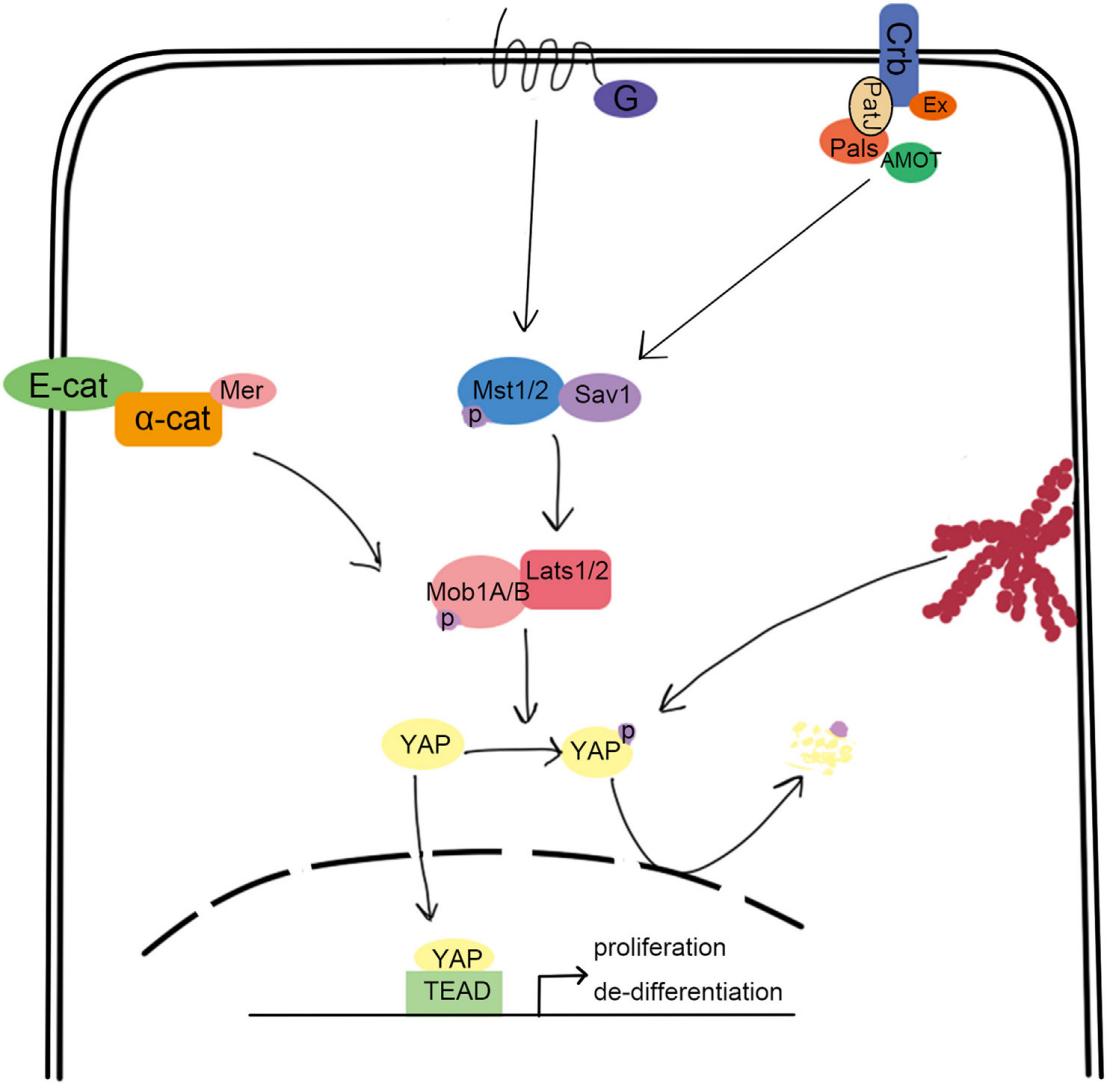
Overview of the canonical hippo signaling pathway. Signals such as mechanical stress, and GPCR can activate Mst1/2, bound with Sav1 through the SARAH domain. The activated Mst1/2 kinases phosphorylate and activate Lats1/2, which can interact with YAP through their respective PPxY and WW domains. Lats1/2 then phosphorylate YAP, which traps it in the cytoplasm upon binding to 14-3-3. This results in the loss of YAP function, which is involved in promoting the transcription of cell proliferation-related genes.

The evolutionarily conserved Hippo pathway was first identified in *Drosophila* and comprises several kinases, adaptor proteins, and transcription factors. Mammalian orthologs for each of the core *Drosophila* Hippo proteins are summarized in Figure 1 and include Mst1/2 (ortholog of Hpo), Salv1 (ortholog of Sav), Lats1/2 (ortholog of Wrts), Mob1A/B (ortholog of Mats), YAP/TAZ (orthologs of Yki), and TEAD1-4 (orthologs of Sd) (1–6). Mst1/2 is a member of the STE20 protein kinase family and contains an N-terminal kinase domain and a C-terminal SARAH domain that allows Mst1/2 to form homo or heterodimers (7). The SARAH domain of Mst1/2 also binds to the scaffolding protein, Salv1, resulting in the phosphorylation of Salv1 and subsequent increase of Mst1/2 activity (8, 9). Mob1A/B is also a substrate of Mst1/2, which binds and promotes the activation of the kinases, Lats1/2 (10). The Lats1/2 PPxY domain binds the WW domain of YAP/TAZ, which allows Lats1/2 to recognize the HXRXXS sequence of YAZ/TAZ and phosphorylate all five serine sites (11–13). This phosphorylation event sequesters YAP/TAZ in the cytoplasm by allowing the 14-3-3 family of regulatory proteins to bind. Subsequently, YAP/TAZ are unable to cooperate with the TEAD transcription factors that promote oncogene transcription and cellular proliferation and are also subjected to degradation (14–17). Therefore, the activation of Hippo induces cell apoptosis by downregulating cell proliferation signaling. Indeed, inactivation of canonical Hippo signaling induces cancer, which is supported by many clinical case reports (18–20).

Apart from cancer and tissue development, Hippo signaling proteins also play an important role in the immune system, including T cell development, survival, trafficking, and activation. Moreover, several recent studies have demonstrated that Mst1/2 regulates T cell biology independently of the canonical Hippo signaling pathway. In the following sections of this article, the relationship between Hippo/Mst1/2 and lymphocyte homeostasis, adhesion, proliferation, apoptosis, and differentiation is discussed. We also discuss why the Hippo signaling may differ in organ systems and the immune system.

## T Cell Development and Peripheral Homeostasis of Peripheral T Cell are Affected by Mst1

T lymphocytes originate from hematopoietic stem cells found in the bone marrow and complete development in the thymus, ultimately developing into mature, CD4^+^, or CD8^+^ SP lymphocytes whose TCRs are restricted by self-MHC molecules and are not auto-reactive. The maturation of T cells in the thymus largely depends on their chemokine-mediated migration from the thymic cortex to the medulla (21). During their migration from the superficial cortex to the inner cortex, CD4^−^CD8^−^ DN thymocytes undergo αβTCR gene rearrangement to develop into CD4^+^CD8^+^ DP cells. Then, DP thymocytes become CD8^+^ or CD4^+^ CD69^hi^ SP cells in response to their respective MHC I or MHC II-restricted cues presented by cTECs present in the cortex, which is called positive selection (22). High-avidity self-antigen (presented by Aire^+^ ICAM-1 ^hi^ mTECs in the medulla)–TCR interactions trigger apoptosis in SP thymocytes, a process called negative selection that induces central immune tolerance. Similarly, the development of Treg in the thymus is partly dependent upon a strong self-ligand signaling (23). Mst1/2 regulate key steps of thymocyte development. The population and distribution of DP thymocytes in *Mst1/2*^−/−^ mice is unchanged (24), likely because Mst1/2 are expressed at low levels in DP thymocytes (25). However, CD69^hi^ CD4^+^ and CD8^+^ SP thymocytes are severely reduced in *Mst1*^−/−^ mice (26), suggesting that Mst1 deficiency impairs the positive selection at the transition from the DP to SP stage. While Dong et al. did not find such a reduction of CD69^hi^ CD4^+^ and CD8^+^ SP thymocytes in *Mst1/2*^−/−^ mice (24). The population of *Mst1*^−/−^ HSA^lo^Vβ5^+^ CD4^+^ SP thymocytes is significantly increased and the number of *Mst1*^−/−^ FoxP3^+^ CD4^+^ Treg in the thymus is decreased, suggesting an additional role for Mst1 in negative selection and Treg development in the thymus.

The interaction between SP thymocytes and mTECs is mediated by LFA-1, which binds ICAM-1 on the mTECs to influence negative selection. *In vitro*, the migration velocity of *Mst1*^−/−^ immature SP CD4^+^ T cells on the ICAM-loaded membrane decreases dramatically when stimulated by CCL21. Moreover, the conjugation between *Mst1*^−/−^ immature CD4^+^ SP and cognate Aire^+^ICAM-1^+^ mTEC is unstable, due to defective ICAM-1/LFA-1 clustering (26). These findings collectively provide evidence that Mst1 plays an important role in LFA-1/ICAM-1-related thymocyte migration and antigen recognition, therefore regulating the T cell development in thymus. How Mst1 regulates LFA function is discussed in a later section.

Mature T lymphocytes continuously migrate from the thymus toward the peripheral lymphoid system. In *Mst1*^−/−^ mice, the number of peripheral T cells is dramatically decreased with an unbalanced distribution of different subsets, including reduced frequency of naïve T cells and increased frequency of CD4^+^/CD8^+^ effector/memory T cells (27). Several factors account for the defects in peripheral T cell homeostasis in *Mst1*^−/−^ mice. First, histological analysis of the thymus showed that mature *Mst1*^−/−^ SP thymocytes accumulate in the perivascular space around the thymus, consistent with inefficient egress of mature lymphocytes (28). Second, *Mst1*^−/−^ naïve T cells have a higher level of proliferation and apoptosis in response to TCR signaling. *Mst1*^−/−^ naïve T cells presumably are activated with a lower threshold stimulation *in vivo* and are deleted thereafter through apoptosis, consistent with reduced frequency of naïve T cells (29). It is also proposed that the high levels of proliferation are due to the dysfunction of *Mst1*^−/−^ Treg cells (discussed more in a later section) (27, 30, 31). How Mst1 regulates the egress of lymphocyte and the apoptosis, proliferation will be illustrated in detail in a later section of this review.

In summary, Mst1 deficiency results in inefficient integrin-dependent thymocyte migration and antigen recognition, leading to failed positive and negative selection and decreased numbers and altered activation of peripheral lymphocytes. Thus, Mst1 has a profound role in shaping immunological responses by influencing thymocyte development and lymphocyte activation.

## Mst1 Associated with LFA-1 Controls the Migration of Lymphocyte

**Figure 2:**
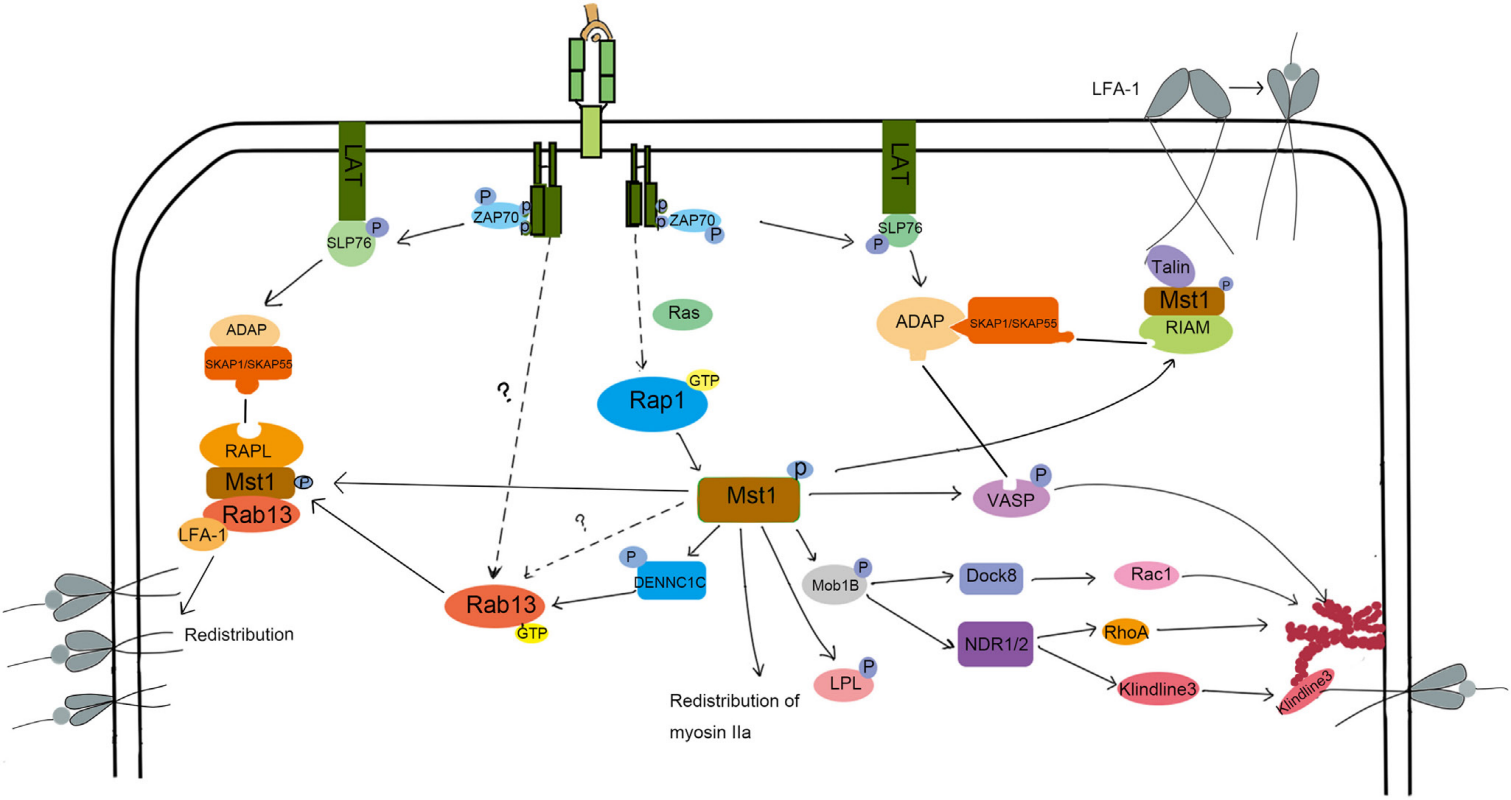
The molecular mechanism of Mst1 regulating the activation and redistribution of LFA-1 and migration. To induce LFA-1 activation and clustering, S1P and CCL21 or TCR signaling activate Mst1 through Rap1. Activated Mst1 then forms a complex with RIAM and Talin and associates with ADAP/SKAP1 to induce LFA-1 activation. Kinlin-3 is also activated during this process and re-distributes to the inner pSMAC, where it interacts with LFA-1 and cytoskeleton to promote the stability of IS. Besides, Mst1 activates Rab13 and forms a RAPL/Mst1/Rab13/LFA-1 complex, directing the LFA-1 redistribution to the ADAP/SKAP1 site. Chemoattract-induced signals also activate Mst1 to induce the phosphorylation of Mob1B, LPL, and VASP to regulate the polymerization of F-actin and cell morphology change, migration, and TCR signal strength.

LFA-1-mediated adhesion to epithelial cells or APC is important for lymphocyte homing and interstitial trafficking. LFA-1 also contributes to the formation of stable immunological synapses in which TCRs bind their specific pMHC, leading to the appropriate activation of T cells. LFA-1 is constitutionally expressed in lymphocytes in a low affinity form. To fulfill its function in directing cell migration and adhesion, LFA-1 undergoes affinity maturation and clustering. The key point in this enhanced affinity is the separation of the αL and β2 domains, which elongates the endoplasmic segments of LFA-1 (32). Mst1 has been demonstrated to be key for LFA-1 functions in T cells. *In vitro, Mst1*^−/−^ T cells are less motile on ICAM-1 when exposed to CCL21 or soluble anti-CD3 antibody. Further, LFA-1 does not undergo affinity maturation in *Mst1*^−/−^ T cells (33). Mechanistically, phosphorylated ITAMs on the TCR recruit and lead to the phosphorylation of ZAP-70. In turn, activated ZAP-70 phosphorylates SLP-76, allowing the SH2 domain of SLP-76 binding to bind ADAP at YDDV sites. The SH3 domain of SKAP1/SKAP55 then binds to the proline-rich region of ADAP (34, 35). This ADAP-SKAP1 interaction inhibits degradation of SKAP1 and allows antigen signals to be transmitted to promote affinity maturation of LFA-1 (36, 37). This pathway is summarized in Figure 2. More recently, ADAP-SKAP/SKAP55 were found to interact with Mst1 in two independent complexes: one that contains RAPL and Rap1; the other that contains RIAM, Rap1, and TALIN or kindlin-3 (38). RAPL is a Rap1 effector protein that directs RAPL/Mst1/Rap1 to the cytomembrane (39). Similarly, RIAM directs the relocalization of RIAM/Mst1/TALIN complexes (40). Then, the FERM domain of TALIN binds to LFA-1-β2 domain, inducing LFA-1 activation. Mst1 deficiency affects the relocation of RAPL/Rap1 and RIAM/Rap1 to the cytomembrane, as well as the activation of LFA-1. On the other hand, the deficiency in RAPL or Rap1 inhibits the activation of Mst1, leading to failed LFA-1-dependent lymphocyte migration (33), which suggests that the structure and kinase activity of Mst1 are both indispensable for LFA-1 activation. How is Mst1 activated in this pathway? It is reported that Rap1 is activated by TCR or chemokine signals, and then recruits SARAH domain-mediated-hetero-dimerization Mst1/RAPL controls Mst1 localization and activation at the membrane, facilitating the activation of LFA-1. Of note, it is reported that kindlin-3 regulated by active Mst1 via NDR1/2 interacts with cytoskeleton at the inner pSMAC to regulate the formation and stability of immunological synapse (41). However, the underlying mechanism needs more explanation. There are some arguments that Mst1 competes with RAPL for binding sites on SARAH domain of SKAP1/SKAP55, which remains to be illustrated.

LFA redistribution, another key factor in LFA-1-dependent migration, is associated with vesicle transportation, in which RapL/Rap1/Mst1 complex plays an important role (Figure 2). After chemokine stimulation in *Mst1*^−/−^ or *RAPL*^−/−^ T cells, the distribution of LFA-1 is dispersed rather than clustered (42, 43). Rab13, a member of the small Ras-related GTP binding protein family, is a key effector of Mst1 in the process of LFA-1 redistribution (44, 45). Immunoprecipitation experiments revealed that Mst1 preferentially interacts with active Rab13. Furthermore, Rab13 has little influence on Mst1 phosphorylation levels, but Mst1 deletion results in a large decrease in Rab13 activity (45). Rab13 is an important effector of Mst1-dependent LFA-1 function, since LFA-1 is unable to cluster at the leading edge or the immune synapse when stimulated by chemokines and antigen in *Rab13*^−/−^ T cells (45). Mechanically, when stimulated with cytokines, Rab13 can be switched into an active, GTP-bound state through Mst1-activated DENNC1C, a GEF for Rab13 (45, 46). In turn, activated Rab13 associates with the LFA and Mst1/RAPL complex, which localizes LFA-1 to the destined leading edge. Indeed, active Rab13 co-localizes with Mst1 in the plasma membrane and peri-nuclear regions, but did not translocate to the leading edge together with LFA-1 until after CXCL12 stimulation. In contrast, LFA-1 does not cluster at the leading edge or promote adhesion and migration of T cells after CXCL12 stimulation when Rab13 is inactive. These results suggest that Rab13 activity is necessary but not sufficient for CXCL12-induced redistribution of LFA-1.

What other Mst1 effectors might promote LFA-1 clustering upon CXCL12 stimulation? Mst1 can also phosphorylate VASP at the serine 157, which allows ADAP to bind to VASP (45). VASP is known to facilitate F-actin polymerization and induce the extension of actin filaments (47). Therefore, Mst1 influences cell shape by controlling F-actin reorganization in a VASP-dependent manner. In doing so, VASP-mediated actin reorganization cooperates with Mst1/Rab13/RAPL to transport the active LFA-1 to the adhesive site, regulating the adhesion and movement of lymphocytes. Mst1 also organizes the immunological synapse by the regulating vesicle transport of the TCR to the cSMAC. Indeed, VSP4, a biomarker of TCR-enriched vesicle (48), and Rab8 or Rab11, proteins that mediate TCR vesicle transport (41, 49), are not enriched at the immunological synapse in Mst1/2-deficient T cells.

In conclusion, Mst1 regulates the activation and redistribution of LFA-1 to affect T cell migration and TCR signaling. The ADAP/SKAP1 complex is an important adaptor that regulates the accurate relocation of RAPL/RAP1/Mst1/Rab13 and RAPL/RAP1/Mst1/TALIN or kindlin-3 complexes to the adhesive site. Rap1/RAPL may be the activator of Mst1 downstream of chemokines like CXCL12, whose physical structure contributes to the stability of the above complex. Further, Mst1 kinase activity facilitates the polymerization of F-actin and vesical transportation of LFA-1 through VASP and Rab13.

## The Role of Mst1 in the Regulation of Cytoskeleton Rearrangement in T Cells

Cell motility is dependent upon the depolymerization and polymerization of actin filaments, which facilitates the cell’s morphology, protrusion, and uropod formation. When adhesion molecules cluster at the leading edge and detach at the trailing edge, the cell body is pulled in the direction of chemokine gradients that induce migration (50). The protein LPL is abundant in lymphocytes, especially in the actin structures called lamellipodium. Mst1 directly phosphorylates LPL at threonine 89, inducing the formation of lamellipodium and promoting cell migration (51). Additionally, Mst1 restricts the distribution of myosin IIa, which co-localizes with F-actin, to affect the cell morphology and promote the localization of LFA-1 to the uropod and trailing edge (52). Polymerization of F-actin is also regulated by localized activation of the Rho family GTPase (53). Experiments have shown that antigen or chemokine-induced actin polymerization and Rho activation are blocked in *Mst1*^−/−^ CD4^+^ T cells. The ability of Mst1 to influence Mob1/Dock8 or Mob1/NDR1/2 activation may participate in controlling Rho activation and actin polymerization as summarized in Figure 2 and discussed below (25, 28).

Chemokines like S1P or CCL21 induce the phosphorylation of Mob1B in an Mst1-dependent manner, and phosphorylated Mob1B interacts with and activates Dock8 (25). Dock8 is a GEF that contains an N-terminal domain and a C-terminal domain, which localize Dock8 to the membrane and regulate its GEF activity, respectively (54). Dock8 then promotes Rac1 activation and subsequent cell migration. Activated Mob1B can also interact with NDR1/2, which allows for the Mst1-dependent phosphorylation of NDR1/2 and induction of Rho GTPase activation. This axis was found to be crucial for facilitating thymocyte egress, homing, and migration (28). It is interesting that the S1P or CCL21-induced phosphorylation of Mob1B is decreased dramatically in *Mst1*^−/−^ T cells but there is no effect on Lats1/2 and NDR1/2 phosphorylation, which indicates that other regulators independent of Mst1 phosphorylate DNR1/2 and activate Rho GTPase (25). These results suggest that Mob1B is a pivotal substrate of Mst1 in chemokine-induced migration. It should be noted that different chemokines seem to activate different small G proteins: CCL21, CCL17, CCL25, CCL19 induce Rac1 activation; S1P induces RhoA activation. As Mst1/2 deficiency leads to impaired Rac1 or RhoA activity, it appears Mst1 plays a role of “relay station” in these signaling pathways to influence cell migration following chemokine stimulation.

In conclusion, Mst1 directly interacts with LPL and restricts the spatial distribution of myosin IIa, regulating the polarization of T cells. Additionally, Mst1 can affect the cytoskeleton rearrangement of chemokine-stimulated T cells by regulating the activity of the Rho family GTPase, in which Mob1 and NDR1/2 phosphorylated by activated Mst1 help to fulfill the activation of Rac1 and RhoA.

## Mst1 and Lymphocyte Proliferation and Apoptosis

As we noted earlier, *Mst1*^−/−^ mice have profound reductions in peripheral T cell numbers and alterations in the antigen-induced apoptosis and proliferation (55). However, there is some controversy as to the role of Mst1 in these processes. Some reports suggested that, upon anti-CD3 and anti-CD28 antibody stimulation, *Mst1*^−/−^ naïve T cells exhibit a higher level of proliferation, while *Mst1*^−/−^ effector/memory T cells do not. However the production of cytokines is enhanced in both naïve and effector/memory T cell subsets when Mst1 is deleted (27). It was found that the expression of Mst1 is nearly 10-fold higher in naïve T cells than effector/memory T cells, suggesting that Mst1 levels dictate a threshold for antigen-induced proliferation of naïve T cells. Therefore, when the restriction is removed upon Mst1 deletion, naïve T cells undergo a rapid proliferation after stimulation with antigen. The next question is how does Mst1 deficiency accelerate the proliferation of naïve T cells after being activated? Thus far, no convincing evidence has been provide, but the canonical substrates of Mst1, Lats1/2, and YAP do not participate in this process as summarized in Table 1 (25, 27).

**Table 1:** The events affected and unaffected in Mst1^−/−^ T cell proliferation.

Events	Affected	Unaffected	Effect
Over-expression of PD-1	√		Higher level of activation-induced Mst1^−/−^ T cell apoptosis
Reduced-expression of FoxO1/3	√		Higher level of Mst1^−/−^ naïve T cell apoptosis
JUK activation	√		Higher level of Mst1^−/−^ effector/memory T cell apoptosis
Erk activation		√	/
P38 activation		√	/
Akt activation		√	/
IkB activation		√	/
Lats1/2 activation		√	/
YAP activation		√	/

In contrast to the above study, other groups have found that antigen-induced proliferation of *Mst1*^−/−^naïve T cells is hindered, with cell cycle progression being partially blocked at the G1/S transition (56). Annexin-V staining in activated total peripheral *Mst1*^−/−^ T cells is upregulated and the expression of Bcl-2 is downregulated (27), which is consistent with increased cell apoptosis contributing to defects in activation-induced proliferation. An important note is that *Mst1*^−/−^ T cells do not exhibit increased apoptosis before activation, suggesting that these defects are related to activation. Activated *Mst1*^−/−^ T cells also have increased expression PD-1 (27), so peripheral CD4^+^ T cell apoptosis could be induced by PD-L1 present in different environments where T cells are inclined for apoptosis. The molecular mechanism of how Mst1 inhibits PD-1 expression on T cells remains to be elucidated. Canonical TCR signaling pathways inducing Lck, ZAP-70, PLCγ1, and Ca^2+^ signaling are not affected in Mst1-deficient T cells. Further, Mst1-deficient T cells have normal activation of Erk, p38, Akt, and phosphorylation of IκB (indicator of NF-κB).

Apart from the high levels of PD-1, *Mst1*^−/−^ naïve T cells have reduced levels of FoxO1/3 (55), transcription factors that facilitate oncogenic gene expression and inhibit cell apoptosis. It is reported that Mst1 increases the expression level of FoxO1/3 by phosphorylating its forkhead domain and therefore inducing its nuclear entrance (57). Another report found that in TCR-stimulated *Mst1*^−/−^ effector/memory T cells, the phosphorylation of Erk, p38, AKT, and IκB are also not altered. However, there is an increased activation of JNK, indicating that JNK hyperactivation might also promote apoptosis in effector/memory *Mst1*^−/−^ T cells (57). It is a paradoxical that Mst1 deficiency leads to enhanced apoptosis in naïve and effector/memory T cells, while the canonical function of Hippo signaling is to inhibit proliferation and induce apoptosis. These differences may be accounted for by a non-canonical function of Mst1/2, as Lats1/2 and YAP functions are not known to be regulated by Mst1 in T cells. These findings indicate that Mst1 signaling pathway is special in lymphocytes and the mechanism it functions in the regulation of lymphocyte proliferation and apoptosis is not clearly illustrated.

In conclusion, *Mst1*^−/−^ naïve CD4^+^ T cells are prone to apoptosis and express higher levels of PD-1 and less FoxO1/3 after stimulation, which results in the defect immune responses. Effector/memory *Mst1*^−/−^ CD4^+^ T cells show a high level of activation-induced apoptosis, which may related with the increased activity of JNK.

## Mst1 and T Cell Differentiation

**Figure 3:**
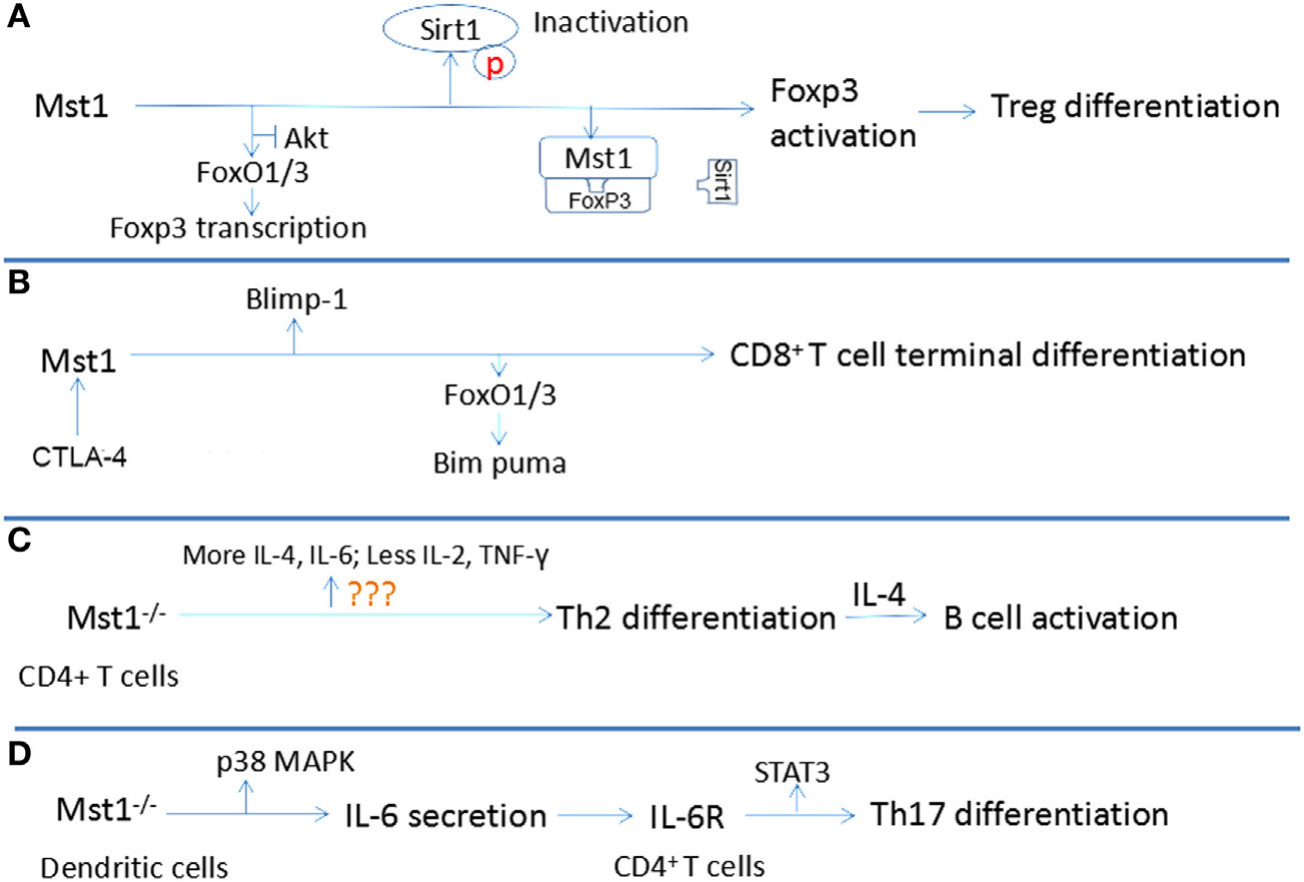
Relationship between Mst1 and T cell differentiation. **(A)** Mst1 promotes the activation of Foxp3 through antagonizing the activity of AKT and Sirt1, inducing the differentiation of regulatory T cell (Treg). **(B)** Combination of CD80 to CTLA-4 activates Mst1, which regulates the activity of Blimp1 and Bim puma, facilitating the terminal differentiation of CD8^+^ T cells. **(C)** Mst1^−/−^ CD4^+^ T cells are inclined to differentiate to T helper cells (Th)2, which induce B cell activation by producing interleukin (IL)-4. **(D)** Mst1^−/−^ dendritic cells secret more IL-6, which regulates Th17 differentiation by agonizing IL-6R of CD4^+^ T cells.

Some researchers stressed that lymphocyte differentiation, but not proliferation and apoptosis, is regulated by the Hippo signaling pathway. The terminal differentiation of Treg and CD8^+^ T cells differentiation, but not proliferation are also dependent upon Mst1 (58). Additionally, the differentiation of naïve CD4^+^ T cells into Th17 and Th2 cells is also regulated by Mst1 Each of these points is discussion in more detail below and are summarized in Figure 3.

The differentiation and activation of Treg cells are restrained in *Mst1*^−/−^ mice, leading to a defect in immune tolerance that results in the over-activation and proliferation of naïve T cells. Indeed, in *Mst1*^−/−^ mice, the number of FoxP3^+^ CD4^+^ Treg is markedly reduced. Similarly, the conversion of naïve CD4^+^ T cells into FoxP3^+^CD4^+^Treg in response to antigen and TGF-β stimulation is also severely reduced in the absence of Mst1. Mechanistically, Mst1 induces FoxP3 expression, the transcription factor that defines the Treg lineage and function by facilitating the expression of CD25 and Nrp1 while suppressing the expression of IL-2 and IFN-γ (59). Mst1 can regulate the expression of FoxP3 at the transcriptional and posttranslational levels. Tao’s group suggests that Mst1 promotes the expression of FoxP3 by antagonizing the activity of AKT, which antagonizes the FoxO1/3a-dependent transcription of FoxP3 (31). The stability and function of FoxP3 requires proper acetylation by lysine acetyl transferases. Therefore, deacetylases like Sirt1, which interacts with FoxP3 and deacetylates FoxP3, inhibit the function of FoxP3 and Treg differentiation (60). Mst1 can protect FoxP3 from being deacetylated by Sirt1, which subsequently enhances the transcriptional activity of FoxP3 to induce Nrp1 and CD25 expression and Treg cell differentiation and function (61). Mst1 exerts its control over Sirt1 by blocking its binding to FoxP3 and by acting in a kinase-dependent manner to inhibit Sirt1 activation. Notably, Mst1 can induce FoxO1/3 nuclear translocation by phosphorylating its forkhead domain in granule neurons (62). FoxP3 is also a forkhead protein, thus suggesting another potential layer by which Mst1 could regulate FoxP3 expression and function.

Further research of *Mst1*^−/−^ Treg found that Mst1 affects the function of Treg by regulating their trafficking and interactions with DC. In secondary lymphoid organ, Treg and naïve T cells move rapidly to recognize the antigen. After the recognition of antigen presented by APC, naïve T cells are arrested and firmly attached with APC, which promotes naïve T cell activation. Meanwhile, Treg move relatively slowly and only temporarily interact with DC, showing a “stop and go” model to exert an immunomodulation function. Adhesion molecule-mediated interactions between APC and Treg are fundamental for Treg functions. The trafficking velocity of *Mst1*^−/−^ Treg is slower than WT Treg, indicating that Mst1-deficient Treg may encounter fewer APC at a slower rate. *In vitro*, conjugate formation between *Mst1*^−/−^ Treg and antigen-specific DC is reduced, which is associated with altered dispersions of LFA-1 and pMHC clusters in the membrane (30). Additionally, Mst1^−/−^ Treg express higher levels of CD86, suggesting that Mst1 regulates the function of Treg through LFA-1-mediated adhesion to DC and not through CD28 co-stimulatory mechanisms. Consistent with the dysfunction of *Mst1*^−/−^ Treg, *Mst1*^−/−^ Treg cannot suppress colitis induced by the adoptive transfer of CD62L^hi^ naïve T cells into healthy mice. In summary, Mst1 deficiency leads to the impaired differentiation and function of Treg, which may contribute to the over-activation and proliferation of naïve CD4^+^ T cells. The underlying mechanism is that Mst1 regulates the immunological synapse formation between Treg and DC. Whether a difference exists in the module of Treg and DC interaction remains to be explored.

Mst1 can also control the terminal differentiation of CD8^+^ T cells. It is known that CD8^+^ T cells will develop into effector CD8^+^ T cells that attack invading pathogens, and memory CD8^+^ T cells that are prepared for a potential future invasion by a previously encountered pathogen. An important point is that CD8^+^ T cells activated by antigen and cytokines should differentiate within a proper time interval after expansion to ensure an effective immune response. If too early, there are not enough effector CD8^+^ T cells to fight against microbes. If too late, microbes have already reached levels out of control. Triggering of the Hippo signaling pathway can certify the accurate regulation of differentiation of activated CD8^+^ T cells. After being activated by antigen and cytokines, the membrane expression of CD80 and CTLA-4 is upregulated and the combination of CD80 and CTLA-4 in activated CD8^+^ T cells will then activate Mst1, leading to the phosphorylation and degradation of YAP through Lats1/2 (58, 63). Degradation of phosphorylated YAP contributes to the higher expression of Blimp-1, which mediates the terminal differentiation of CD8^+^ T cells (64). Moreover, CTLA-4-deficient CD8^+^ T cells do not downregulate YAP protein levels or upregulate Blimp-1 expression, demonstrating a key role for the CTLA-4 signaling axis in regulating canonical Hippo signaling and the induction of Blimp-1 in CD8^+^ T cells. It is also reported that, in primary CD8^+^ T cells, Mst1 deficiency leads to the impaired expression of FoxO3a, which induces the expression of the pro-apoptotic protein Bim. This dysregulation contributes to the excessive proliferation and delayed differentiation of primary CD8^+^ T cells in the absence of Mst1 (65). The probability of CD80/CTLA-4 combination is proportional to the cell density. Since the volume is fixed, the activation of Mst1 is proportional to the activated cell numbers, which makes it possible for Mst1 to control the timed terminal differentiation of CD8^+^ T cells. In summary, interaction between activated CD8^+^ T cells mediated by CD80 and CTLA-4 can induce the activation of Mst1, leading to the degradation of phosphorylated Yap and the upregulation of FoxO3a and Blimp-1 to promote their terminal differentiation.

Activated *Mst1*^−/−^ T cells produce lower levels of the Th1-related cytokines IL-2 and IFN-γ and increased levels of the Th2 cytokine IL-4, indicating that *Mst1*^−/−^ naïve CD4^+^ T cells are inclined to differentiate toward Th2 cells. This biased differentiation may create an IL-4-rich environment which contributes to the activation, proliferation, and differentiation of plasmacytes, accounting for the hypergammaglobulinemia of *Mst1*^−/−^ mice. Indeed, *Mst1*^−/−^ naïve T cells can promote the activation of B cells and plasma cell differentiation, resulting in a higher serum level of IgG, IgA and IgE (66). CD40-CD40L interactions on respective B cells and T cells provide a crucial second signal for B cell activation; however, *Mst*^−/−^ CD4^+^T cells do not express aberrant levels of CD40L.

Mst1 can also indirectly influence IL-17-producing Th17 cell differentiation. DC can modulate the differentiation of Th17 by producing IL-6, which is dependent upon Mst1 (67). Mechanistically, Mst1 deficiency in DC leads to increased LPS-induced p38 activation, which increases the production of IL-6. The IL-6-rich environment induces more IL-6R expression in the membrane of CD4^+^ T cells. Subsequently, IL-6R stimulation by IL-6 activates STAT3, a key transcription factor that promotes naïve CD4^+^ T cell differentiation into Th17 cells (68). Taken together, these studies demonstrate that Mst1 can shape the adaptive immune response by regulating the differentiation of naïve T cells into specific subsets and Treg cell development and function.

## Comparison of Hippo Signaling Between Other Tissues and the Immune System

As proposed at the beginning of the article, canonical Hippo signaling regulates organ size by inhibiting tissue cell proliferation and promoting differentiation. Depletion of Mst1/2 in liver cells results in hepatomegaly and liver cancer, with an increased population of atypical ductal cells that bear characteristics of hepatic progenitors (69). At the cellular level, *Mst1/2*^−/−^ converts the cuboidal hepatocytes to atypical cells that behave like hepatic progenitors. The molecular mechanism underlying this process is that Mst1/2 deletion enhances the level of YAP through the canonical Hippo signal pathway. YAP translocates to the nucleus and interacts with TEAD1-4, which leads to the re-programming of adult hepatocytes and results in the de-differentiation of mature hepatocytes. Additionally, it is reported that the NOTCH pathway, known as a vital regulator of biliary cell fates during embryogenesis, is one of effectors of activated YAP. It is of significant interest to determine the other effectors of YAP in this Hippo signaling pathway as a means to limit liver cancer development and induce liver regeneration. Unlike the liver, the heart tissue does not undergo self-renewal when injured. However, cardiomyocytes deficient for Mst1/2 show an improved regenerative ability because YAP-dependent gene transcription linked to F-actin polymerization and cell cycle progression is increased (70). By comparison, *Mst1/2*^−/−^ T cells have impaired F-actin polymerization, cell mobility, and cell proliferation with cell cycle progression being partially blocked at the G1/S transition.

Why does the Hippo signal pathway function differently between immune system and organs systems like the liver and heart? One explanation may lie in the different upstream receptor pathways that trigger Hippo signaling. In the canonical Hippo signaling pathway as shown in Figure 1, cell polarity, cell contact, extracellular mechanical signals, rearrangement of cytoskeleton, and GPCRs can regulate the activation of the Hippo pathway, allowing Hippo to regulate organ size (6, 44–46). Mst1/2 can be phosphorylated by the adjunction molecules, Mer and Ex, in polar cells to promote cell differentiation and inhibit cell proliferation. GPCR signals induced by SIP inhibit Mst1/2 phosphorylation, which is reported to be related with the F-actin remodeling, leading to YAP activation. However, this situation is different in lymphocytes, where TCR or chemokine stimulation (including S1P) promotes Mst1 phosphorylation by Rap1 (or other unknown molecules) to positively regulate the rearrangement of actin and LFA-1 clustering to induce T cell adhesion. As for the regulation of proliferation and apoptosis of lymphocytes, no uniform conclusion has been reached. Reports regarding how Mst1 is activated in lymphocytes are limited. Whether cell–cell interactions between lymphocytes or some other factors reflecting the expansion of lymphocytes can affect the activity of Mst1 is important for us to understand the role of Hippo signaling in regulation of lymphocyte proliferation and differentiation. In addition to potential differences in upstream regulators, Mst1/2 can regulate the activation of downstream targets in the immune system that are different than canonical Hippo targets activated in tissues, like liver. For instance, with the exception of CD80 and CTLA-4 noted above, Lats1/2 and YAP activity are not regulated by Mst1 in lymphocytes.

Taken together, triggering of the Hippo pathway is very different between lymphocytes and traditional tissues, such as the liver and heart. The latter is regulated by F-actin mediated Hippo activation, while the former is Mst1-mediated actin polymerization. As for the regulation of proliferation and apoptosis in lymphocytes, more research is needed to address the precise role of Mst1 in this process. Therefore, the differences of Mst1 signal between the immune system and traditional tissues may due to its lack of understanding, or may be due to the differences between adherent cells in the environment.

## Concluding Remarks

The conserved Hippo signaling pathway discovered in *Drosophila* plays essential roles in the generation of organs and development of cancer. Because of the special way it perceives signals, it can sense the mechanical cues outside the cell, including cell–cell interactions, which allows it to participate in the regulation of organ growth. Recent studies have also highlighted key roles for the mammalian Hippo kinases for the protection from aberrant immune reactions that can cause T cell-related inflammatory infiltration of multiple organs and auto-antibodies production. The function of Mst1 in the process of lymphocyte migration, development, activation, proliferation, and differentiation is gradually becoming more understood. However, many details in these processes are not clear. Except for RAPL/Rap1, are there other molecules regulating the activation of Mst1 in TCR and chemoattractant signaling pathways? In the immune system, can the cytoskeleton affect the activation of Mst1, as has been reported for the Hippo signaling pathway (6, 71–73)? The molecular mechanisms of Mst1 in lymphocyte proliferation and apoptosis are still not fully understood, but are important to address because the canonical Hippo signal pathway is a key regulator of these processes in organ systems. Although Mst1/2 do not usually appear to signal via Lats1/2 or YAP in immune cells, is it possible that there are homologous molecules of Lats1/2, Mob1A/B, and YAP/TAZ in immune system participating the survival signal of lymphocytes? Additionally, what others subsets of T cells are tightly associated with Mst1 and how does Mst1 function in these subsets? In summary, studies to date suggest primarily non-canonical roles for the Mst1 signaling pathway is different in immune system. Decoding the activators and effectors of Mst1 in the immune system will provide cues toward the treatment of some autoimmune disease and immunodeficiency disorders and improve the development of novel drug therapies.

## Nomenclature

**Table:** 

APC	T cell-antigen-presenting cell
Akt	protein kinase B
ADAP	adhesion and degranulation promoting adapter protein
Blimp-1	PR domain zinc finger protein 1
GPCR	G protein-coupled receptor
Bcl-2	B-cell lymphoma 2
cTECs	cortex thymic epithelial cells
CCL21	chemokine (C-C motif) ligand 21
CTLA-4	cytotoxic T-lymphocyte-associated protein 4
CD28	cluster of differentiation 28
CD80	cluster of differentiation 80
CD4	cluster of differentiation 4
CD8	cluster of differentiation 8
DP	double positive
Dock8	dedicator of cytokinesis 8
DENNC1C	differentially expressed in normal and neoplastic cell domain 1C
DC	dendritic cell
Erk	extracellular signal-regulated kinases
Foxo1/3	forkhead box protein O1/3
FoxP3	forkhead box P3
GEF	guanine nucleotide exchange factor
GPCRs	G-protein-coupled receptors
Hpo	Hippo
IkB	nuclear factor kappa-light-chain-enhancer of activated B cells
IL2	interleukin 2
IL-6	interleukin 6
IL-17	interleukin 17
IL-4	interleukin 4
IFN-e	interferon gamma
Lck	lymphocyte-specific protein tyrosine kinase
ZAP-70	zeta-chain-associated protein kinase 70
LPL	actin-bundling protein L plastin
JNK	c-Jun N-terminal kinases
LFA-1	lymphocyte function-associated antigen 1
Mst1/2	mammalian sterile 20-like kinase
Mob1A/B	Mps one binder kinase activator-like 1
Mats	Mob as tumor suppressor
MFI	mean fluorescence intensity
mTECs	medullary thymic epithelial cells
NDR1/2	nuclear Dbf2-related 1/2, homolog of Lats
Nrp1	neuropilin-1
PPxY	Pro-Pro-Xaa-Tyr
pMHC	peptide-majorhistocompatibility complex
PD-1	programmed cell death protein 1
p38	mitogen-activated protein kinases
PLCγ1	phosphoinositide phospholipase C
Rac1	Ras-related C3 botulinum toxin substrate 1
RhoA	Ras homolog gene family, member A
Rab13	a member of small Ras-related GTP binding protein family
RAPL	regulator for cell adhesion and polarization enriched in lymphoid tissue (also known as RASSF5)
SP	single positive
Sav	salvador
Sd	scalloped
Salv	salvador homolog 1
Lats1/2	large tumor suppressor homolog ½
STE20	sterile 20
SARAH	Sav/Rassf/Hpo
S1P	sphingosine-1phosphate
SKAP1/SKAP5	Src kinase-associated phosphoprotein 1/55
SLP-76	lymphocyte cytosolic protein 2
TEAD1-4	TEA domain family member 1 to 4
Treg	regulatory T cells
TCR	T cell receptor
Th1	T helper cells 1
Th2	T helper cells 2
Th17	T helper cells 17
TGF-beta	transforming growth factor beta 1
VASP	vasodilator-stimulated phosphorylation
Wts	Warts
Yki	Yorkie
YAP/TAZ	Yes-associated protein/transcriptional coactivator with PDZ-binding motif
14-3-3	a family of conserved regulatory molecules that are expressed in all eukaryotic cells

## Author Contributions

CL organized the article. JC wrote the draft. YJ, DK, LY, ZY, ZP, XL, YW, and QG revised the draft. JL drew the figures. RM and YZ edited the language and figures.

## Conflict of Interest Statement

We declare that the research was conducted in the absence of any commercial or financial relationships that could be construed as a potential conflict of interest.

## References

[1] Justice RW Zilian O Woods DF Noll M Bryant PJ. The *Drosophila* tumor suppressor gene warts encodes a homolog of human myotonic dystrophy kinase and is required for the control of cell shape and proliferation. Genes Dev (1995) 9:534–46. 10.1101/gad.9.5.534 7698644

[2] Boedigheimer M Laughon A. Expanded: a gene involved in the control of cell proliferation in imaginal discs. Development (1993) 118:1291–301. 8269855 10.1242/dev.118.4.1291

[3] Watson KL Justice RW Bryant PJ. Drosophila in cancer research: the first fifty tumor suppressor genes. J Cell Sci Supplement (1994) 18:19–33. 10.1242/jcs.1994.Supplement_18.4 7883789

[4] Lai ZC Wei X Shimizu T Ramos E Rohrbaugh M Nikolaidis N Control of cell proliferation and apoptosis by mob as tumor suppressor, mats. Cell (2005) 120:675–85. 10.1016/j.cell.2004.12.036 15766530

[5] Dong J Feldmann G Huang J Wu S Zhang N Comerford SA Elucidation of a universal size-control mechanism in drosophila and mammals. Cell (2007) 130:1120–33. 10.1016/j.cell.2007.07.019 17889654 PMC2666353

[6] Zhao B Li L Wang L Wang CY Yu J Guan KL. Cell detachment activates the Hippo pathway via cytoskeleton reorganization to induce anoikis. Genes Dev (2012) 26:54–68. 10.1101/gad.173435.111 22215811 PMC3258966

[7] Scheel H Hofmann K A novel interaction motif, SARAH, connects three classes of tumor suppressor. Curr Biol (2003) 13:R899–900. 10.1016/j.cub.2003.11.007 14654011

[8] Pantalacci S Tapon N Leopold P. The salvador partner hippo promotes apoptosis and cell-cycle exit in drosophila. Nat Cell Biol (2003) 5:921–7. 10.1038/ncb1051 14502295

[9] Hwang E Cheong HK Ul Mushtaq A Kim HY Yeo KJ Kim E Structural basis of the heterodimerization of the MST and RASSF SARAH domains in the hippo signalling pathway. Acta Crystallogr D Biol Crystallogr (2014) 70:1944–53. 10.1107/S139900471400947X 25004971 PMC4089488

[10] Praskova M Xia F Avruch J. MOBKL1A/MOBKL1B phosphorylation by MST1 and MST2 inhibits cell proliferation. Curr Biol (2008) 18:311–21. 10.1016/j.cub.2008.02.006 18328708 PMC4682548

[11] Huang J Wu S Barrera J Matthews K Pan D. The hippo signaling pathway coordinately regulates cell proliferation and apoptosis by inactivating yorkie, the drosophila homolog of YAP. Cell (2005) 122:421–34. 10.1016/j.cell.2005.06.007 16096061

[12] Edgar BA. From cell structure to transcription: hippo forges a new path. Cell (2006) 124:267–73. 10.1016/j.cell.2006.01.005 16439203

[13] Shi Z Jiao S Zhou Z. Structural dissection of hippo signaling. Chin J Biochem Biophys (2015) 47:29–38. 10.1093/abbs/gmu107 25476203

[14] Avruch J Zhou D Fitamant J Bardeesy N Mou F Barrufet LR. Protein kinases of the Hippo pathway: regulation and substrates. Semin Cell Dev Biol (2012) 23:770–84. 10.1016/j.semcdb.2012.07.002 22898666 PMC3489012

[15] Zhao B Wei X Li W Udan RS Yang Q Kim J Inactivation of YAP oncoprotein by the Hippo pathway is involved in cell contact inhibition and tissue growth control. Genes Dev (2007) 21:2747–61. 10.1101/gad.1602907 17974916 PMC2045129

[16] Zhao B Ye X Yu J Li L Li W Li S TEAD mediates YAP-dependent gene induction and growth control. Genes Dev (2008) 22:1962–71. 10.1101/gad.1664408 18579750 PMC2492741

[17] Zhou Y Huang T Cheng AS Yu J Kang W To KF. The TEAD Family and Its Oncogenic Role in Promoting Tumorigenesis. Int J Mol Sci (2016) 17:E138. 10.3390/ijms17010138 26805820 PMC4730377

[18] Yimlamai D Fowl BH Camargo FD. Emerging evidence on the role of the Hippo/YAP pathway in liver physiology and cancer. J Hepatol (2015) 63:1491–501. 10.1016/j.jhep.2015.07.008 26226451 PMC4654680

[19] Ma Y Yang Y Wang F Wei Q Qin H. Hippo-YAP signaling pathway: a new paradigm for cancer therapy. Int J Cancer (2015) 137:2275–86. 10.1002/ijc.29073 25042563

[20] You B Yang YL Xu Z Dai Y Liu S Mao JH Inhibition of ERK1/2 down-regulates the Hippo/YAP signaling pathway in human NSCLC cells. Oncotarget (2015) 6:4357–68. 10.18632/oncotarget.2974 25738359 PMC4414195

[21] Witt CM Raychaudhuri S Schaefer B Chakraborty AK Robey EA. Directed migration of positively selected thymocytes visualized in real time. PLoS Biol (2005) 3:e160. 10.1371/journal.pbio.0030160 15869324 PMC1088277

[22] Takahama Y. Journey through the thymus: stromal guides for T-cell development and selection. Nat Rev Immunol (2006) 6:127–35. 10.1038/nri1781 16491137

[23] Sakaguchi S Yamaguchi T Nomura T Ono M. Regulatory T cells and immune tolerance. Cell (2008) 133:775–87. 10.1016/j.cell.2008.05.009 18510923

[24] Dong Y Du X Ye J Han M Xu T Zhuang Y A cell-intrinsic role for Mst1 in regulating thymocyte egress. J Immunol (2009) 183:3865–72. 10.4049/jimmunol.0900678 19692642

[25] Mou F Praskova M Xia F Van Buren D Hock H Avruch J The Mst1 and Mst2 kinases control activation of rho family GTPases and thymic egress of mature thymocytes. J Exp Med (2012) 209:741–59. 10.1084/jem.20111692 22412158 PMC3328371

[26] Ueda Y Katagiri K Tomiyama T Yasuda K Habiro K Katakai T Mst1 regulates integrin-dependent thymocyte trafficking and antigen recognition in the thymus. Nat Commun (2012) 3:1098. 10.1038/ncomms2105 23033074

[27] Zhou D Medoff BD Chen L Li L Zhang XF Praskova M The Nore1B/Mst1 complex restrains antigen receptor-induced proliferation of naive T cells. Proc Nat Acad Sci U S A (2008) 105:20321–6. 10.1073/pnas.0810773105 PMC2600581 19073936

[28] Tang F Gill J Ficht X Barthlott T Cornils H Schmitz-Rohmer D The kinases NDR1/2 act downstream of the hippo homolog MST1 to mediate both egress of thymocytes from the thymus and lymphocyte motility. Sci Signal (2015) 8:ra100. 10.1126/scisignal.aab2425 26443704

[29] Krammer PH Arnold R Lavrik IN. Life and death in peripheral T cells. Nat Rev Immunol (2007) 7:532–42. 10.1038/nri2115 17589543

[30] Tomiyama T Ueda Y Katakai T Kondo N Okazaki K Kinashi T. Antigen-specific suppression and immunological synapse formation by regulatory T cells require the Mst1 kinase. PLoS One (2013) 8:e73874. 10.1371/journal.pone.0073874 24040101 PMC3767606

[31] Du X Shi H Li J Dong Y Liang J Ye J Mst1/Mst2 regulate development and function of regulatory T cells through modulation of Foxo1/Foxo3 stability in autoimmune disease. J Immunol (2014) 192:1525–35. 10.4049/jimmunol.1301060 24453252

[32] Dustin ML Bivona TG Philips MR. Membranes as messengers in T cell adhesion signaling. Nat Immunol (2004) 5:363–72. 10.1038/ni1057 15052266

[33] Katagiri K Imamura M Kinashi T. Spatiotemporal regulation of the kinase Mst1 by binding protein RAPL is critical for lymphocyte polarity and adhesion. Nat Immunol (2006) 7:919–28. 10.1038/ni1374 16892067

[34] Wang H Wei B Bismuth G Rudd CE. SLP-76-ADAP adaptor module regulates LFA-1 mediated costimulation and T cell motility. Proc Nat Acad Sci U S A (2009) 106:12436–41. 10.1073/pnas.0900510106 19617540 PMC2710989

[35] Wang H McCann FE Gordan JD Wu X Raab M Malik TH ADAP-SLP-76 binding differentially regulates supramolecular activation cluster (SMAC) formation relative to T cell-APC conjugation. J Exp Med (2004) 200:1063–74. 10.1084/jem.20040780 15477347 PMC2211848

[36] Wang H Liu H Lu Y Lovatt M Wei B Rudd CE. Functional defects of SKAP-55-deficient T cells identify a regulatory role for the adaptor in LFA-1 adhesion. Mol Cell Biol (2007) 27:6863–75. 10.1128/MCB.00556-07 17646386 PMC2099233

[37] Raab M Wang H Lu Y Smith X Wu Z Strebhardt K T cell receptor “inside-out” pathway via signaling module SKAP1-RapL regulates T cell motility and interactions in lymph nodes. Immunity (2010) 32:541–56. 10.1016/j.immuni.2010.03.007 20346707 PMC3812847

[38] Kliche S Worbs T Wang X Degen J Patzak I Meineke B CCR7-mediated LFA-1 functions in T cells are regulated by 2 independent ADAP/SKAP55 modules. Blood (2012) 119:777–85. 10.1182/blood-2011-06-362269 22117043

[39] Raab M Smith X Matthess Y Strebhardt K Rudd CE. SKAP1 protein PH domain determines RapL membrane localization and Rap1 protein complex formation for T cell receptor (TCR) activation of LFA-1. J Biol Chem (2011) 286:29663–70. 10.1074/jbc.M111.222661 21669874 PMC3191007

[40] Menasche G Kliche S Chen EJ Stradal TE Schraven B Koretzky G. RIAM links the ADAP/SKAP-55 signaling module to Rap1, facilitating T-cell-receptor-mediated integrin activation. Mol Cell Biol (2007) 27:4070–81. 10.1128/MCB.02011-06 17403904 PMC1900018

[41] Kondo N Ueda Y Kita T Ozawa M Tomiyama T Yasuda K NDR1-Dependent regulation of kindlin-3 controls high-affinity LFA-1 binding and immune synapse organization. Mol Cell Biol (2017) 37:e424–416. 10.1128/MCB.00424-16 28137909 PMC5376635

[42] Katagiri K Katakai T Ebisuno Y Ueda Y Okada T Kinashi T. Mst1 controls lymphocyte trafficking and interstitial motility within lymph nodes. EMBO J (2009) 28:1319–31. 10.1038/emboj.2009.82 19339990 PMC2683056

[43] Kanda I Nishimura N Nakatsuji H Yamamura R Nakanishi H Sasaki T. Involvement of Rab13 and JRAB/MICAL-L2 in epithelial cell scattering. Oncogene (2008) 27:1687–95. 10.1038/sj.onc.1210812 17891173

[44] Katagiri K Maeda A Shimonaka M Kinashi T. RAPL, a Rap1-binding molecule that mediates Rap1-induced adhesion through spatial regulation of LFA-1. Nat Immunol (2003) 4:741–8. 10.1038/ni950 12845325

[45] Nishikimi A Ishihara S Ozawa M Etoh K Fukuda M Kinashi T Rab13 acts downstream of the kinase Mst1 to deliver the integrin LFA-1 to the cell surface for lymphocyte trafficking. Sci Signal (2014) 7:ra72. 10.1126/scisignal.2005199 25074980

[46] Yoshimura S Gerondopoulos A Linford A Rigden DJ Barr FA. Family-wide characterization of the DENN domain Rab GDP-GTP exchange factors. J Cell Biol (2010) 191:367–81. 10.1083/jcb.201008051 20937701 PMC2958468

[47] Doppler HR Bastea LI Lewis-Tuffin LJ Anastasiadis PZ Storz P. Protein kinase D1-mediated phosphorylations regulate vasodilator-stimulated phosphoprotein (VASP) localization and cell migration. J Biol Chem (2013) 288:24382–93. 10.1074/jbc.M113.474676 23846685 PMC3750140

[48] Choudhuri K Llodra J Roth EW Tsai J Gordo S Wucherpfennig KW Polarized release of T-cell-receptor-enriched microvesicles at the immunological synapse. Nature (2014) 507:118–23. 10.1038/nature12951 24487619 PMC3949170

[49] Finetti F Paccani SR Riparbelli MG Giacomello E Perinetti G Pazour GJ Intraflagellar transport is required for polarized recycling of the TCR/CD3 complex to the immune synapse. Nat Cell Biol (2009) 11:1332–9. 10.1038/ncb1977 19855387 PMC2837911

[50] Ridley AJ Schwartz MA Burridge K Firtel RA Ginsberg MH Borisy G Cell migration: integrating signals from front to back. Science (2003) 302:1704–9. 10.1126/science.1092053 14657486

[51] Xu X Wang X Todd EM Jaeger ER Vella JL Mooren OL Mst1 kinase regulates the actin-bundling protein l-plastin to promote T cell migration. J Immunol (2016) 197:1683–91. 10.4049/jimmunol.1600874 27465533 PMC4992580

[52] Xu X Jaeger ER Wang X Lagler-Ferrez E Batalov S Mathis NL Mst1 directs Myosin IIa partitioning of low and higher affinity integrins during T cell migration. PLoS One (2014) 9:e105561. 10.1371/journal.pone.0105561 25133611 PMC4136924

[53] Gomez TS Billadeau DD. T cell activation and the cytoskeleton: you can’t have one without the other. Adv Immunol (2008) 97:1–64. 10.1016/S0065-2776(08)00001-1 18501768

[54] Meller N Merlot S Guda C. CZH proteins: a new family of Rho-GEFs. J Cell Sci (2005) 118:4937–46. 10.1242/jcs.02671 16254241

[55] Du X Yu A Tao W. The non-canonical Hippo/Mst pathway in lymphocyte development and functions. Chin J Biochem Biophys (2015) 47:60–4. 10.1093/abbs/gmu112 25487919

[56] Salojin KV Hamman BD Chang WC Jhaver KG Al-Shami A Crisostomo J Genetic deletion of Mst1 alters T cell function and protects against autoimmunity. PLoS One (2014) 9:e98151. 10.1371/journal.pone.0098151 24852423 PMC4031148

[57] Choi J Oh S Lee D Oh HJ Park JY Lee SB Mst1-FoxO signaling protects Naive T lymphocytes from cellular oxidative stress in mice. PLoS One (2009) 4:e8011 10.1371/journal.pone.0008011 19956688 PMC2776980

[58] Thaventhiran JE Hoffmann A Magiera L de la Roche M Lingel H Brunner-Weinzierl M Activation of the Hippo pathway by CTLA-4 regulates the expression of Blimp-1 in the CD8+ T cell. Proc Natl Acad Sci U S A (2012) 109:E2223–9. 10.1073/pnas.1209115109 22745171 PMC3421161

[59] Fontenot JD Gavin MA Rudensky AY. Foxp3 programs the development and function of CD4+CD25+ regulatory T cells. Nat Immunol (2003) 4:330–6. 10.1038/ni904 12612578

[60] van Loosdregt J Vercoulen Y Guichelaar T Gent YY Beekman JM van Beekum O Regulation of treg functionality by acetylation-mediated Foxp3 protein stabilization. Blood (2010) 115:965–74. 10.1182/blood-2009-02-207118 19996091

[61] Li J Du X Shi H Deng K Chi H Tao W. Mammalian Sterile 20-like Kinase 1 (Mst1) enhances the stability of Forkhead Box P3 (Foxp3) and the function of regulatory T Cells by modulating Foxp3 acetylation. J Biol Chem (2015) 290:30762–70. 10.1074/jbc.M115.668442 26538561 PMC4692206

[62] Lehtinen MK Yuan Z Boag PR Yang Y Villen J Becker EB A conserved MST-FOXO signaling pathway mediates oxidative-stress responses and extends life span. Cell (2006) 125:987–1001. 10.1016/j.cell.2006.03.046 16751106

[63] Pentcheva-Hoang T Egen JG Wojnoonski K Allison JP. B7-1 and B7-2 selectively recruit CTLA-4 and CD28 to the immunological synapse. Immunity (2004) 21:401–13. 10.1016/j.immuni.2004.06.017 15357951

[64] Rutishauser RL Martins GA Kalachikov S Chandele A Parish IA Meffre E Transcriptional repressor Blimp-1 promotes CD8(+) T cell terminal differentiation and represses the acquisition of central memory T cell properties. Immunity (2009) 31:296–308. 10.1016/j.immuni.2009.05.014 19664941 PMC2783637

[65] Yasuda K Ueda Y Ozawa M Matsuda T Kinashi T. Enhanced cytotoxic T-cell function and inhibition of tumor progression by Mst1 deficiency. FEBS Lett (2016) 590:68–75. 10.1002/1873-3468.12045 26787462

[66] Park E Kim MS Song JH Roh KH Lee R Kim TS. MST1 deficiency promotes B cell responses by CD4+ T cell-derived IL-4, resulting in hypergammaglobulinemia. Biochem Biophys Res Commun (2017) 489:56–62. 10.1016/j.bbrc.2017.05.094 28527887

[67] Jones GW McLoughlin RM Hammond VJ Parker CR Williams JD Malhotra R Loss of CD4+ T cell IL-6R expression during inflammation underlines a role for IL-6 trans signaling in the local maintenance of Th17 cells. J Immunol (2010) 184:2130–9. 10.4049/jimmunol.0901528 20083667

[68] Li C Bi Y Li Y Yang H Yu Q Wang J Dendritic cell MST1 inhibits Th17 differentiation. Nat Commun (2017) 8:14275. 10.1038/ncomms14275 28145433 PMC5296641

[69] Yimlamai D Christodoulou C Galli GG Yanger K Pepe-Mooney B Gurung B Hippo pathway activity influences liver cell fate. Cell (2014) 157:1324–38. 10.1016/j.cell.2014.03.060 24906150 PMC4136468

[70] Morikawa Y Zhang M Heallen T Leach J Tao G Xiao Y Actin cytoskeletal remodeling with protrusion formation is essential for heart regeneration in hippo-deficient mice. Sci Signal (2015) 8:ra41. 10.1126/scisignal.2005781 25943351 PMC4442128

[71] Yu FX Zhao B Panupinthu N Jewell JL Lian I Wang LH Regulation of the Hippo-YAP pathway by G-protein-coupled receptor signaling. Cell (2012) 150:780–91. 10.1016/j.cell.2012.06.037 22863277 PMC3433174

[72] Wada K Itoga K Okano T Yonemura S Sasaki H. Hippo pathway regulation by cell morphology and stress fibers. Development (2011) 138:3907–14. 10.1242/dev.070987 21831922

[73] Mo JS Yu FX Gong R Brown JH Guan KL. Regulation of the Hippo-YAP pathway by protease-activated receptors (PARs). Genes Dev (2012) 26:2138–43. 10.1101/gad.197582.112 22972936 PMC3465735

